# Neuroprotective Effects of Curcumin in Neurodegenerative Diseases

**DOI:** 10.3390/foods13111774

**Published:** 2024-06-05

**Authors:** Giuseppe Genchi, Graziantonio Lauria, Alessia Catalano, Alessia Carocci, Maria Stefania Sinicropi

**Affiliations:** 1 Dipartimento di Farmacia e Scienze della Salute e della Nutrizione, Università della Calabria, Arcavacata di Rende, 87036 Cosenza, Italy; giuseppe.genchi@unical.it (G.G.); glauria@unical.it (G.L.); s.sinicropi@unical.it (M.S.S.); 2 Dipartimento di Farmacia-Scienze del Farmaco, Università degli Studi di Bari “Aldo Moro”, 70125 Bari, Italy; alessia.carocci@uniba.it

**Keywords:** neurodegenerative diseases, nanoparticles, *Curcuma longa*, bioavailability

## Abstract

Curcumin, a hydrophobic polyphenol extracted from the rhizome of *Curcuma longa*, is now considered a candidate drug for the treatment of neurological diseases, including Parkinson’s Disease (PD), Alzheimer’s Disease (AD), Huntington’s Disease (HD), Multiple Sclerosis (MS), Amyotrophic Lateral Sclerosis (ALS), and prion disease, due to its potent anti-inflammatory, antioxidant potential, anticancerous, immunomodulatory, neuroprotective, antiproliferative, and antibacterial activities. Traditionally, curcumin has been used for medicinal and dietary purposes in Asia, India, and China. However, low water solubility, poor stability in the blood, high rate of metabolism, limited bioavailability, and little capability to cross the blood–brain barrier (BBB) have limited the clinical application of curcumin, despite the important pharmacological activities of this drug. A variety of nanocarriers, including liposomes, micelles, dendrimers, cubosome nanoparticles, polymer nanoparticles, and solid lipid nanoparticles have been developed with great success to effectively deliver the active drug to brain cells. Functionalization on the surface of nanoparticles with brain-specific ligands makes them target-specific, which should significantly improve bioavailability and reduce harmful effects. The aim of this review is to summarize the studies on curcumin and/or nanoparticles containing curcumin in the most common neurodegenerative diseases, highlighting the high neuroprotective potential of this nutraceutical.

## 1. Introduction

**Figure 1:**
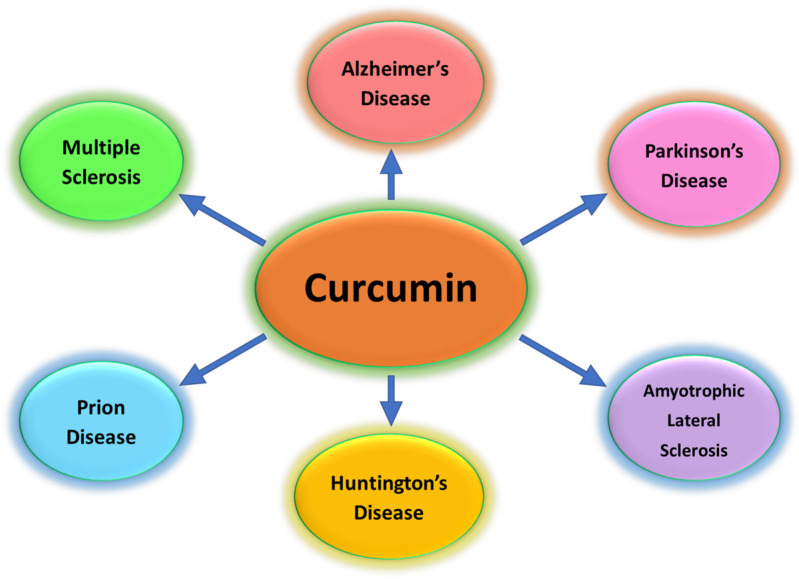
Application of curcumin in neurological diseases.

The brain is a metabolically very active organ, which needs a large quantity of oxygen to function. In the mitochondria of all cells (even brain cells), oxidation-reduction reactions occur in the presence of oxygen to synthesize adenosine triphosphate (ATP). At the same time, reactive oxygen species (ROS) are produced, thanks to the redox reactions of oxidizable substrates, oxidative phosphorylation reactions, and scarce presence of endogenous antioxidants. The high levels of free radicals in the central nervous system (CNS) are involved in inflammatory processes and in the pathogenesis and progression of neurodegenerative processes [1]. Neurodegenerative diseases (NDs) are disorders characterized by a progressive deterioration of the function and structure of the neuron population in the CNS [2]. These age-related brain disorders worsen many physical activities, including balance and motor coordination, speech ability, and respiratory and cardiovascular functions [3]. NDs like Alzheimer’s Disease (AD), Parkinson’s Disease (PD), Huntington’s Disease (HD), Amyotrophic Lateral Sclerosis (ALS), Multiple Sclerosis (MS), dementia with Lewy bodies (DLB), corticobasal degeneration, prion disease, and progressive supranuclear palsy [4,5,6] represent a serious threat to human health. They are becoming increasingly widespread, also because the number of elderly people has increased in recent years. NDs are characterized by several natural changes that lead to neuronal dysfunction and death. These changes lead to mitochondrial dysfunctions, oxidative and nitrosative stress, apoptosis, and uncontrolled neuronal inflammation. There are also mutations in mitochondrial DNA, abnormalities in the activities of the complexes of the mitochondrial transport chain, and incomplete inhibition of ATP production with overproduction of free radicals that damage membrane phospholipids, proteins, DNA, and RNA. They not only reduce patient life expectancy and life quality, but also take a heavy toll on family members, also increasing the financial burden to patients and family. However, they are largely untreatable. The aerobic mitochondrial metabolism generates toxic by-products like ROS, reactive nitrogen species (RNS), and free radicals [7]. Furthermore, genetic disorders and exogenous pollutants, such as radiations and smoke, increase ROS levels. ROS allow for the quick acceptance and donation of electrons by interacting with and damaging proteins/enzymes, lipids and phospholipids, and DNA and RNA, thus affecting their structures and functions. Cells are protected by a synergistic activity of several different antioxidants, which stabilize or deactivate free radicals. Antioxidants, which scavenge free radicals and chelate redox metals, may be endogenous organic molecules or exogenous compounds taken in the diet [8]. Antioxidants are classified based on their activity (enzymatic or non-enzymatic), chemical–physical properties, and chemical structures (polyphenols, carotenoids, flavonoids, terpenes, etc.). In the past years, several natural antioxidant compounds have been proven as therapeutic strategies to slow or halt the progression of neurodegenerative diseases [9,10]. Curcumin, extracted from the rhizome of *Curcuma longa*, has been demonstrated to exhibit beneficial and neuroprotective effects and has been suggested as a promising candidate in several NDs (Figure 1). Data from the literature demonstrate curcumin’s ability to modulate different signaling pathways involved in the development of neurodegenerative diseases, such as nuclear factor-erythroid 2-related factor 2 (Nrf2), serine/threonine kinase AKT, and transcription factor nuclear factor-kB (NF-kB) [11,12]. Furthermore, curcumin may be useful in the treatment of cardiovascular, hepatic, and renal diseases. In addition, curcumin possesses a wide variety of biological actions, showing antitumor, pulmonoprotective, antithrombotic, antidiabetic, and anti-inflammatory properties, as well as antibacterial, antifungal, antihypertensive, anti-inflammatory, and antihepatotoxic effects [13,14,15,16,17,18]. It may also act as an antiviral by inhibiting the replication in a wide range of viruses, being also suggested as a promising therapy for the treatment and prevention of COVID-19 [19,20]. However, the therapeutic activity of curcumin is limited due to its low water solubility, fast biological metabolism, low bioavailability, and limited blood–brain barrier (BBB) permeation. The encapsulation of this drug into nanoparticles and nanovectors may be useful to overcome low solubility, poor bioavailability, and inability to penetrate the BBB and neuronal membranes.

## 2. Curcumin: Bioavailability and Metabolism

**Figure 2:**
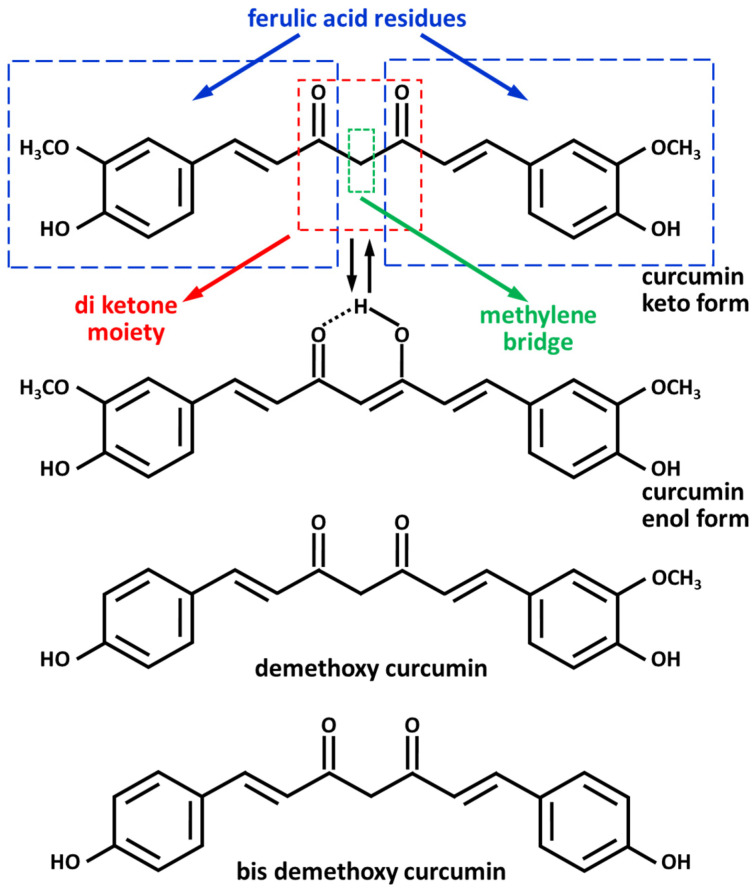
Curcumin shows keto–enol tautomerism. The keto form is present in neutral and acidic conditions (pH = 3–7), while the enol form is stable in alkalin solutions (pH ≥ 8). Demethoxycurcumin and bisdemethoxycurcumin are two other components in *Curcuma longa*.

Curcumin, a golden-colored non-toxic polyphenol, extracted from turmeric (*Curcuma longa* L.) has been used in traditional Indian and Chinese medicine and cuisine for a long time. Turmeric is mainly composed of curcuminoids, including curcumin (77%), demetoxycurcumin (17%), and bisdemetoxycurcumin (6%). Curcumin [1,7-bis(4-hidroxy-3-methoxyphenyl)-1,6-eptadiene-3,5-dione] is a polyphenol (molecular weight = 368.3 g/mol) that is made up of two ferulic acid residues linked by a methylene group (Figure 2) [21].

Curcumin is a hydrophobic compound that is insoluble in water. It has low solubility in hydrocarbon solvents and is easily soluble in ethanol, methanol, acetonitrile, and ethyl acetate [21]. It exists in tautomeric keto–enol conformations, and the relative concentration of keto–enol forms depends on the temperature, solvent polarity, and pH. In neutral and acidic conditions (pH 3–7), curcumin is found in the keto form acting as a proton donor; in basic conditions (pH ≥ 8), it is more stable than the enol form, which behaves as an electron donor [21,22]. The phenolic group of the molecule is a significant electron donor for antioxidant activities, anti-inflammatory capacities, and anticancer effects [23]. The aromatic groups of this compound provide curcumin hydrophobicity with poor water solubility. Aromatic *o*-methoxyphenol, α,β-unsaturated β-diketo moiety, and seven carbon linkers are important for the activity of curcumin. In fact, the *o*-methoxyphenol and the methylenic hydrogens are responsible for the antioxidant activity of curcumin, which reacts with ROS-donating hydrogen atoms. Curcumin interacts with several biomolecules through covalent and non-covalent bindings. The β-diketone moiety covalently binds the -OH, -SH, and -SeH groups of proteins; in addition, the β-diketone groups form chelates with heavy transition metals (Cu^2+^, Zn^2+^, and Fe^3+^) reducing their toxicity [24]. The curcumin methylene group of β-diketone is a potent scavenger of superoxide anion (O_2_^•−^), hydroxyl radical (^•^OH), hydrogen peroxide (H_2_O_2_), nitric oxide (NO^•^), hydroperoxyl radical (HOO^•^), peroxyl radical (ROO^•^), and ROS. Curcumin also enhances the activity of several antioxidant enzymes like catalase, glutathione peroxidase, superoxide dismutase (SOD), heme oxygenase-1, and nicotinamide adenine dinucleotide phosphate (NADPH) oxidase [25,26]. However, curcumin has limited bioavailability and is metabolized in the liver by aldo–keto reductase, in addition to having low water solubility (0.4 μg/mL at normal gastric pH 1.5–4.0) and limited BBB permeability. To solve these problems, various systems have been developed, including nanocarrier preparations for curcumin delivery, such as liposomes, micelles, solid lipid nanoparticles (SLNs), liquid crystalline nanoparticles (LCNs), polymeric nanoparticles, cell membrane nanocarriers, and cyclodestrin [27,28,29]. To determine the pharmacokinetics and pharmacodynamics of curcumin, Ravindranath and Chandrasekhara (1981, 1982) [30,31] administered ^3^H-curcumin to rats, showing that curcumin undergoes several metabolic biochemical transformations. In fact, in the intestine, curcumin suffers a stepwise reduction in the presence of reductases forming dihydrocurcumin, tetrahydrocurcumin, esahydrocurcumin, and octahydrocurcumin [24]. In the presence of UDP glucuronosyltransferase, the reduced curcumin derivatives are transformed into curcumin, dihydrocurcumin, tetrahydrocurcumin, hexahydrocurcumin, and octahydrocurcumin glucuronide derivatives. In addition, curcumin forms curcumin sulphate by reacting with phenol sulfotransferase enzyme. When curcumin is taken orally, it is transformed into curcumin glucuronide and curcumin sulfate, while curcumin administered intraperitoneally undergoes reduction reactions [24].

## 3. Curcumin Nanoparticles

Curcumin is currently being studied for its anti-inflammatory, antioxidant, antimicrobial, anticancer, and antihyperglycemic activities, in addition to its essential involvement in neurological disorders and cardiovascular disease [32,33,34,35,36,37]. Despite such medical applications and treatments, curcumin has a limited therapeutic value due to its poor water solubility, slow absorption rate, rapid metabolism, and poor bioavailability, thus restricting its clinical use. Nanoparticle delivery systems are generally used to enhance physicochemical stability, water solubility, and targeting. However, exogenous nanoparticles can be easily removed by the immune system. In this context, the usefulness of curcumin-loaded biomimetic nanomedicines prepared with cell membranes and extracellular vesicles has been exploited [38]. Several activities were enhanced by using nanoparticles containing curcumin. Recently, cell membrane-coated carriers have been explored for targeted drug delivery by camouflaging particles to evade the immune system and to enhance their targeted delivery in chemotherapy. The preparation of curcumin-loaded porous poly(lactic-co-glycolic acid) (PLGA) nanoparticles (NPs) and surface modification with red blood cell membranes was carried out to accelerate drug release [39]. Curcumin-loaded exosomes were used in an in vivo mouse model study to successfully cross the BBB and deliver the drug to treat malignant glioma in the brain [40]. A significant amelioration of ulcerative colitis and apical periodontitis was obtained by encapsulating curcumin into CXCR4 cell membrane vesicles through physical entrapment. The obtained cell membrane vesicles induced higher M2 macrophage polarization and showed anti-inflammatory effects [41,42]. The anti-inflammatory efficacy of curcumin-loaded nanoparticles has been recently evidenced, as they were able to reduce the expression of Nf-κb, TGF-β1, IL-6, and IL-1β in lipopolysaccharide-inflamed cells, thus suggesting these nanoparticles as possible candidates for the targeted anti-inflammatory therapy of atherosclerosis [43]. For the treatment of neurological disorders, including AD, PD, HD, ALS, and MS, intelligent nanoformulation curcumin delivery systems, such as liposomes, micelles, solid lipid particles, and nanofibers, can be used; their size must be <200 nm to easily cross the BBB. Chemical modification reactions are carried out to obtain a more lipophilic system to increase the uptake across the BBB by replacing hydrophilic groups with lipophilic groups and reducing polar groups. Additionally, the surface of nanoparticles can be functionalized through chemical reactions in which the targeting ligands are coupled to bind receptors and transporters in the delivery of curcumin to brain cells [44]. The favorite mode of delivery in the brain can be obtained by intracerebral injection, intranasal route, intravenous injection, oral administration, and aerosol-based techniques [45].

### 3.1. Curcumin Nanoparticles in Parkinson’s Disease

PD, which is a slowly progressive neurodegenerative disorder with the loss of dopaminergic neurons in the substantia nigra pars compacta, is characterized by dopamine deficiency in the brain [46]. Moreover, the formation of α-synuclein (a small protein of about 20 kDa) aggregation is a common finding in PD. Dopamine plays an important role in motor coordination, muscle rigidity, reduced voluntary movement, sleep disturbance, memory cognition, and depression especially in people over age 60. Epidemiologic studies suggest that the occurrence of PD is higher in Caucasian populations than in African and Asian ones. Pringsheim et al. (2014) [47] emphasized a big difference in the prevalence of PD between Asia (646/100,000) and North America, Europe, and Australia (1601/100,000) in members of the population between 70 and 79 years of age. Aggregations are detrimental to dopaminergic neurons, causing the formation of Lewy bodies [48]. These aggregates in non-pathological conditions are usually demolished by lysosomes and proteasome complexes. However, defects in these scavenging pathways are usual in PD, causing a further proliferation of these aggregates. Treatment of PD with levodopa (L-3,4-dihydroxyphenylalanine), as a prodrug of dopamine, restores the function of depleted dopamine in the striatum. Long-term treatment with this compound after several months to years develops undesirable effects in patients, like dyskinesia, gastrointestinal disorders, chest pain, hives, and weakness. With a limitation in the use of levodopa, other clinical strategies have been adopted to improve dopamine release like dopamine agonist (DA), monoamine oxidase type B (MAO-B) inhibitor, β-blocker, and adamantine; however, prolonged treatment with these compounds leads to the development of undesirable harmful reactions. Curcumin is emerging as a potential candidate for innovative strategies as an adjuvant therapy in PD, especially using nanopreparations. Siddique et al. (2013) [49] studied the effect of an alginate–curcumin nanopreparation on the oxidative stress and brain death in a transgenic *Drosophila* model observing the effect on the climbing ability of the PD model flies and lipid peroxidation in the brain of the flies. After supplementing the diet of the flies with an alginate–curcumin nanopreparation for 24 days, a significant loss of climbing ability of the PD model flies and a reduction in the oxidative stress and apoptosis were observed in the brain of the flies in the PD *Drosophila* model. Bollimpelli et al. (2016) [50] prepared lactoferrin nanoparticle curcumin (LF-NP-cur) by sol–oil chemistry to protect SK-N-SH cells against rotenone-induced neurotoxicity. Moreover, the LF-NP-cur reduced the level of ROS induced by rotenone as well as the aggregation of α-synuclein in SK-N-SH cells. Kundu and coauthors (2016) [51] loaded curcumin and piperine into glyceryl monooleate (GMO) nanoparticles. A GMO-NP-Pip-Cur nanopreparation hindered the aggregation of α-synuclein into oligomers and fibrils; in addition, the anti-apoptotic activity of this preparation was studied without showing any cytotoxicity. In vitro, GMO-NP-Pip-Cur diminished the motor dysfunction thus reducing apoptosis and oxidative stress, while improving autophagy. In a PD mouse model, in the presence of this piperine–curcumin nanopreparation, an increased density of tyrosine hydroxylase (TH) positive neuron was observed, as well as the ability of this dual drug loaded with nanoparticles to cross the BBB. On the other hand, the research group of Taebnia et al. 2016 [52] developed a drug carrier based on mesoporous silica nanoparticles functionalized with [3-(2-aminoethylamino)propyltrimethoxy]silane loaded with curcumin. These silica nanoparticles possess high drug-loading and good entrapment efficiency. In addition, α-synuclein species, interacting with this nanosilica formulation, significantly inhibited the fibrillation process, but did not affect the cytotoxic properties of the formed fibrils. Rakotoarisoa and collaborators (2019) [53] studied the neuroprotective effect of curcumin and fish oil, rich in ω-3 polyunsaturated fatty acids, and loaded spongosome and cubosome nanoparticles in human SH-SY5Y cells, showing the neuroprotective activity against ROS accumulation and cell death induced by H_2_O_2_. Sookhaklari and coauthors (2019) [54] suggested that BSA-based nanocurcumin developed a neuroprotective effect against cell death induced by 6-hydroxydopamine (6-OHDA) in SH-SY5Y cells.

### 3.2. Curcumin Nanoparticles in Alzheimer’s Disease

The most common early sign of AD is the difficulty in remembering recent events. As the disease progresses, symptoms include speech problems from uttering single words to complete loss of speech, disorientation, loss of motivation, behavioral issues, and self-neglect. Due to declining health conditions, patients are excluded from family and society. Gradually, bodily functions are lost, ultimately leading to death. AD is a disease due to the accumulation of abnormally folded β-amyloid (Aβ) protein (made up of small peptides formed from about 40 amino acids) that form amyloid-insoluble plaques and tau protein in the neurofibrillary tangles in the brain. Aβ protein is a fragment derived from the amyloid-beta precursor protein (APP) thanks to the action of γ- and β-secretases. Medications used to treat AD consist of acetylcholinesterase inhibitors, like tacrine, rivastigmine, metrifonate, and donepezil, and a noncompetitive NMDA (N-Methyl-D-Aspartate) receptor antagonist as memantine. These inhibitors were able to maintain the level of acetylcholine and reverse the symptoms of short-term memory loss and confusion. However, these medications do not yield truly positive results; rather, they reduce the progression of the disease [55]. The keto–enol ring of curcumin covalently reacts with the aromatic rings of Aβ, inhibiting the aggregation of senile plaques and promoting the decomposition of these plaques [56]. Furthermore, the two polar -OH groups located at the two ends of the molecule, which form hydrogen bonds with the amino acids present in the polar pocket of the Aβ protein, are also important for destabilizing the β-sheets. There is several experimental evidence that curcumin in AD treatment has regulated different pathways, such as inflammation reduction, neurogenesis activation, and Aβ inhibition [57]. It has been reported that curcumin has shown antiamyloidogenic activity, not only inhibiting Aβ aggregation, but also disaggregating existing ones. In 2017, Barbara and collaborators [58] encapsulated curcumin in biodegradable PLGA nanoparticles modified with glycosylated hepta-peptide, called g7, to cross the BBB and studied its toxicity, targeting the delivery and the biological activity in an in vitro primary hippocampal cell culture model of AD. The obtained nanoparticles reduced the level of oxidative stress and inflammation and enhanced Aβ disaggregation. Fan et al. (2018) [59] prepared a brain-targeted nanoparticle PLGA-block-poly-ethylene glycol conjugated with B6 peptide and loaded with curcumin (PLGA-PEG-B6-CUR). The authors administered this nanopreparation in in vitro mice hippocampal neuronal HT22 cells and ex vivo APP/PS1 A1 transgenic mice. The in vivo assay using dynamic light scattering, red blood cell lysis, and thromboelastographic methods indicated that these nanoparticles showed good bio-safety and high bioavailability. The results from the Morris water maze obtained with APP/PS1 A1 transgenic mice proved that this PLGA-PEG-B6-Cur nanoencapsulation exceptionally increased the spatial learning and memory ability of the transgenic mice. In addition, treatment with these nanoparticles reduced the Aβ level and tau phosphorylation. Drug delivery of curcumin nanoencapsulation in AD therapy was developed by Huo et al. (2019) [60]. The transgenic 5XFAD mice were treated with a curcumin-loaded selenium-poly-lactic-co-glycolic acid (Se-PLGA) nanospherical formulation. Curcumin-loaded Se-PLGA nanospheres decreased Aβ in mice brains and were able to cure memory deficiency in transgenic mice.

### 3.3. Curcumin Nanoparticles in Huntington’s Disease

Huntington’s Disease is an autosomal dominant inherited degenerative disorder with massive neuronal degeneration, motor and cognitive impairment, and psychiatric symptoms. This neurodegenerative disease arises from a trinucleotide repeat disorder, due to the length of a repeated section of the Huntington gene (*HTT*), placed on chromosome 4p163, exceeding a normal range.

The Huntington gene contains a repetitive sequence of three bases CAG (cytosine–adenine–guanine) that aids in protein aggregation throughout the brain, therefore damaging neurons. CAG is the three-letter genetic code for the amino acid glutamine (Q) resulting in the production of a long chain of glutamine known as the polyglutamine tract or polyQ tract. Generally, there are about 36–39 glutamine repeats in the polyQ region, which leads to the production of the cytoplasmic huntingtin protein [61,62]. It is characterized by involuntary movements, abnormalities in gait and posture, obsessive–compulsive behavior, memory loss, and psychiatric dysfunction. In addition, oxidative stress, mitochondrial dysfunction, inflammation, and transcriptional dysregulation play an important role in the progression of HD. During its early stages, the expansion of polyglutamine is the main cause of striatum degeneration; with progression of the disease, neurodegeneration diffuses to other regions of the brain, such as the cortex, hippocampus, thalamus, hypothalamus, nucleus accumbens, and substantia nigra. The analysis of postmortem brain tissues in HD patients showed that alterations in the Ca^++^ homeostasis, the electron transport chain, and the mitochondrial Krebs cycle were observed [63,64]. Because the electron transport chain and the mitochondrial tricarboxylic acid cycle are involved in the production of ATP, tissues with high energy production and consumption are more subjected to oxidative stress. Moreover, the high membrane phospholipid concentration along with the high energy consumption in brain cells led to a greater probability of oxidative damage. During dysfunction in the mitochondrial transport chain, superoxide anions damage phospholipids, proteins, and DNA. Sandhir et al. (2014) [65] used curcumin-encapsulated solid lipid nanoparticles (Cur-SLNs) in rats to abolish HD-induced by 3-nitro propionic acid (3NP) and to enhance its oral bioavailability. The results obtained with this nanocurcumin preparation showed that Cur-SLN administration in rats revealed an important enhancement in the activity of mitochondrial complexes, such as NADH dehydrogenase, succinate dehydrogenase, cytochrome oxidase, mitochondrial F_1_F_0_ synthase, and levels of cytochrome a, b, c_1_, and c [66]. In addition, a considerable decrease in mitochondrial swelling, lipid peroxidation, and ROS was observed in rats treated with Cur-SLN, while the levels of glutathione (GSH) and superoxide dismutase were increased. In a study of Gharaibeh et al. (2020) [67], eleven-month-old transgenic YAC 128 HD mice, which had the full-length human *HTT* with 128 CAG repeats, were treated orally with 100 mg/kg of solid lipid curcumin particles (SLCPs) every other day for eight weeks to test the efficacy of this formulation in reducing deficits. After eight weeks, the mice were euthanized and the brain was stained using the Golgi–Cox method to study the length and the number of dendrites. This research showed that curcumin solid lipid particle treatment increased dendritic arborization and the density of dendritic spines and reduced learning memory deficits and post-synaptic density protein-95 (PSD-95).

### 3.4. Curcumin Nanoparticles in Amyotrophic Lateral Sclerosis

ALS is a rare and lethal neurodegenerative disorder, which leads to the progressive loss of upper and lower motor neurons, i.e., the nerve cells in the spinal cord and brain, which control voluntary muscle movement and breathing. Upper motor neurons in the brain send signals to the lower motor neurons, resulting in the stimulation of muscles in walking, talking, eating, and breathing. Two kinds of ALS are known: sporadic ALS, which affects about 90–95% of people with this disease, and appears without a clear cause, and familial ALS (FALS), which affects 5–10% of people with ALS. FALS is caused by changes to a gene that is transmitted to children. About 30–40% of all familial cases are caused by a defect in the chromosome 9 open reading frame 72 (C9orf72) gene, while the other 15–20% of familial cases result from a mutation in the SOD1 gene. The protein C9orf72 is found in many regions of the brain and in the cytoplasm of neurons. The genetic abnormality leading to neurodegeneration consists of an exposure of a stretch of six nucleic acids of DNA of the C9orf72 gene. The sequence GGGGCC (guanine–cytosine) in healthy individuals is repeated approximately 20 times, while in individuals affected by ALS, it is repeated up to 1600 times. The enzyme SOD1 is involved in the synthesis of copper–zinc superoxide dismutase1. SOD1 is responsible for destroying free superoxide radicals in the body. A cytoplasmic isozyme and a mitochondrial isozyme are known; both convert superoxide radicals into oxygen and hydrogen peroxide. ALS does not affect human intelligence and the ability to think, see, or hear. When motor neurons degenerate, the brain loses control of voluntary movements, such as walking, talking, and chewing. Early symptoms include muscles cramps, muscles weakness affecting arms and legs, and tight and stiff muscles. As the disease progresses, people with ALS develop problems with chewing food, drooling, speaking or forming words, maintaining weight, and getting enough nutrients. In addition, because it is a progressive disease, it will lead to paralysis and ultimately to death. Unfortunately, there is no known cure for ALS except for riluzole. In this regard, the use of mesenchymal stromal cells (MSCs) as a therapy for ALS has been addressed for many years in clinical studies [68,69,70]. Mesenchymal stromal cells (MSCs) are the spindle-shaped plastic-adherent cells that can be isolated from human adult tissue, such as bone marrow, heart, brain, spleen, kidneys, adipose tissue, and umbilical cord, which can be expanded in vitro. MSCs can release high quantities of molecules with immunomodulatory properties and are able to differentiate into osteogenic, adipogenic, and myogenic cells that can migrate and repair damaged tissues [71]. Actually, MSCs are used in the treatment of ALS thanks to the improvement of neural protection and their ability to replace dead motor neurons in the spinal cord. Curcumin-loaded inulin-D-α-tocopherol succinate micelles (INVITE-MC), which can penetrate the damaged tissues due to their dimensions (about 7 nm diameter), were used to enhance the therapeutic effect of MSCs. This system ensures accurate drug targeting, because the drug is able to reach damaged tissues, while protecting the incorporated curcumin against a premature release [72,73,74].

### 3.5. Curcumin Nanoparticles in Multiple Sclerosis 

MS is a chronic inflammatory autoimmune disease, which damages the insulating materials of the nerve fibers (myelin) in the brain and spinal cord. The damage of the nerve cells acts negatively on the nervous system’s ability to transmit signals that control cognitive, emotional, and physical abilities. Other symptoms include fatigue, vision loss, double vision, blurry vision, muscle weakness and spams, loss of coordination, and bladder control issues [75]. The pathological inflammation results in demyelination, reduced remyelination, and decreased axon survival and neuronal death. Multiple sclerosis occurs in two forms: individual relapse or gradual progression. In the relapsing form of this disease, symptoms may completely disappear, although some permanent neurological problems arise again over several years. In the progressive form, the functions of the body slowly deteriorate with a gradual worsening of the disability. There are many possible causes that can increase the risk of developing multiple sclerosis, including autoimmune disorders (pernicious anemia, thyroid disease, and psoriasis), infectious agent (Epstein–Barr virus), and environmental and genetic factors. There is no cure for this disease; in any case, treatments are known to help modify the course of the disease and manage the symptoms. Thanks to its antioxidant and anti-inflammatory properties, curcumin may be considered an excellent therapeutic candidate for multiple sclerosis; in fact, curcumin antioxidative activity decreases neuronal death [76]. Curcumin shows important neuroprotective results in multiple sclerosis cases through its antioxidant, anti-proliferative, and anti-inflammatory mechanisms due to enzyme interaction, (cyclooxygenase-2, hemeoxygenase-1, (NAD(P)H oxidoreductase, lipoxygenase, and xanthine oxidase), proteins (caspase3/9, prostaglandin E2, C-reactive protein, and B-cell lymphoma protein 2 (Bcl-2)), inflammatory cytokines (interleukins, interferon gamma, tumor necrosis factor alpha, and chemokine ligands), and transcriptional factors (activating protein-1, nuclear factor-kappa B, and nuclear factor erythroid 2-related factor). Natarajan and Bright (2002) [77] used animal models of MS characterized by experimental autoimmune encephalomyelitis (EAE) produced by injecting mice with myelin. These EAE mice were treated parentally with 50–100 μg of curcumin. Mice treated with curcumin showed no MS symptoms, while those not treated with the drug were paralyzed and then died. The authors showed that this result is explained by the inhibition of IL-12 that caused myelin plaque damage. In another study on Lewis rats with induced EAE, Mohajeri and coauthors (2015) [78] demonstrated that polymerized nanocurcumin particles (12.5 mg/kg) produced the remyelination of neurons by repairing neuron myelin sheaths. Curcumin dendrosomal nanoparticles were used by Motavaf et al. (2020) [79] in the process of remyelination and oligodendrogenesis in vitro and in an animal model or in demyelination induced by the toxin cuprizone. Cuprizone is a copper chelator and mitochondrial toxin; therefore, its intake lowers mitochondrial activity and activates an oxidative stress response, leading to oligodendrocyte death. The scientists showed that dendrosomal curcumin nanoparticles enhanced in vitro oligodendrogenesis in a dose-dependent manner. The nanoparticle preparation induced in vivo remyelination and the dendrosomal nanoparticles also enhanced the remyelination ability of transplanted neural stem cells, promoting their oligodendrogenesis capacity.

### 3.6. Curcumin Nanoparticles in Prion Disease

The word prion, coined by S.B. Prusiner (1982, 1998) [80,81], is derived from the words protein and infection, and it is an acronym for proteinaceous infectious particles. This protein produces fatal degenerative diseases of the CNS in mammals. Prions are the agents responsible for the human Creutzfeldt–Jakob Disease and Kuru Disease and fatal familial insomnia; prions may also cause diseases in animals, such as scrapie in sheep, bovine spongiform encephalophaty in cattle, feline spongiform encephalophaty in cats, and chronic wasting disease in elks and deer. The prion protein is present in two forms: PrPC (normal cellular prion protein) and PrPSc (scrapie infectious form). PrPC and PrPSc are different only in terms of their secondary and tertiary structures. The PrPC form presents a predominant α-helical structure, while the PrPSc form is formed of both α-helix and β-pleated sheets. The β-pleated sheets in abnormal proteins interact between protein molecules forming insoluble plaques, which disrupt the normal tissue structure. Prion aggregates produce characteristic holes (vacuoles) within the CNS, which make for a spongy neuronal architecture. The physiological role of prions is poorly understood. Research by Abbott (2010) [82] on mice showed that the cleavage of prion protein in peripheral nerves results in the activation of myelin repair in Schwann cells, while the lack of the prion protein causes demyelination in the same cells. In addition, Lathe and Darlix (2017) [83] showed that PrP can play an important role in innate immunity. Moreover, the prion protein exhibits antimicrobial activity, also inhibiting the replication of several viruses; this protein interacts also with the Aβ peptide in Alzheimer’s Disease. PrP and Aβ can both penetrate the membranes, bind nucleic acids, and have antiviral properties with the antimicrobial peptide LL-37. It is known that the incubation time for prion disease is relatively long (10 to 20 years), but when the symptoms manifest, the disease progresses rapidly, resulting in brain damage and then death. Neurodegenerative symptoms are dementia, convulsion, coordination dysfunction, and behavioral or personality changes. Prions are glycoproteins (molecular mass of 35–38 kDa) found in the cell membranes of nerve tissues and in the membranes of hematopoietic cells, the precursors of blood stream cells. The PrPSc prion protein can convert normal PrPC protein into the infectious isoform, thus changing its conformation. When humans eat tainted beef derived from animals with mad cow disease, they can be affected by Creutzfeldt–Jakob Disease. PrPScs are stable and resistant to denaturation by proteases, temperature, and chemical agents; namely, they cannot be removed by sanitation or cooking. Caughey and coauthors (2003) [84] have shown that yellow-dye curcumin inhibits in vitro the prion fibril formation in scrapie-infected neuroblastoma (scNB) cells, and partially inhibits the cell-free conversion of PrPc to PrPSc. Lin and coresearchers (2013) [85] demonstrated that curcumin is a promising molecule in inhibiting the accumulation of the prion PrPrSc. To analyze the effect of curcumin on fibril formation, the authors studied the effect of the molecule in a cell-free system on mouse prion protein, showing a notable reduction in prion fibril formation. Furthermore, their research demonstrated that curcumin treatment of mouse neuroblastoma cells (N2a) protects N2a cells from apoptosis and reverses the level of ROS caused by incubation with prior amyloid fibrils. The diazo dye Congo red, structurally related to curcumin, binds to amyloid fibrils and may lower the accumulation of the abnormal PrPSc in scrapie-infected cells and inhibit PrPSc formation in a cell-free system. It was thought to be a medication to treat prion disease. However, it is carcinogenic and is not able to cross the BBB. For these reasons, Congo red cannot be used as specific medication to treat prion disease, unlike curcumin.

## 4. Conclusions

The CNS is metabolically highly active, sensible to ROS damage, and liable to develop degenerative conditions depending on oxidative stress. Neuroinflammation and neuronal damage are believed to be a major cause of neurological diseases. Symptoms and damage-related disorders can be treated with various approaches. Several small molecules or natural compounds have been approved by the Food and Drug Administration (FDA) and the European Medicine Agency (EMA) to treat and inhibit these misfolded protein aggregations. Despite the clinical availability of several drugs and phytochemicals for treating neurodegenerative diseases (PD, AD, HD, MS, ALS, prion disease, and brain tumor), none of them can be successfully used in clinical therapy, and they can be utilized only as palliative therapies. These cures provide temporary symptom relief and are also associated with severe side effects; in addition, most of these drugs are expensive. Several studies have shown that phytochemicals, like flavonoids, polyphenols, terpenes, and alkaloids, can alleviate neurological disorders in vivo, in vitro, and in clinical treatments. Curcumin, a polyphenol herbal drug obtained from *Curcuma longa*, has the necessary properties to fight several neurodegenerative diseases. Curcumin is a safe, natural, and economic compound, but its biomedical potential is limited due to its low solubility in water, incomplete absorption by the gut, poor bioavailability, fast metabolism and elimination from the body, and little BBB penetration capacity in significant quantities. Moreover, due to its natural origin, curcumin can become part of a nutritional plan, and can be administered for prolonged periods without harmful effects. To overcome any inconvenience, various curcumin delivery systems have been developed based on nanoparticles like liposomes, micelles, solid lipid nanoparticles, polymeric nanoparticles, and inorganic nanocarriers. Nanocarriers can enhance the water solubility of this polyphenolic compound, improve its cellular uptake, and increase its antioxidant and anti-inflammatory properties. Curcumin may be incorporated into cell membrane-camouflaged nanoparticles, which are biomimetic nanoparticles that combine the unique functionalities of cellular membranes and the engineering versatility of synthetic nanomaterials for the efficient delivery of therapeutic agents. The surface of nanoparticles can be modified and functionalized with brain-specific ligands for effective BBB penetration to bring curcumin to specific sites within the brain and neuronal membranes. Curcumin is regularly found in Indian and Chinese cuisine, and its benefits for human health have been evidenced through pharmacological and clinical research. In conclusion, seeing as curcumin is inexpensive and shows no side effects, it can be considered a strong candidate as a neuroprotective agent.

## Data Availability

No new data were created or analyzed in this study. Data sharing is not applicable to this article.

## References

[1] Halliwell B. (2001). Role of free radicals in the neurodegenerative diseases: Therapeutic implications for antioxidant treatment. Drugs Aging.

[2] Golpich M., Amini E., Mohamed Z., Azman Ali R., Mohamed Ibrahim N., Ahmadiani A. (2017). Mitochondrial dysfunction and biogenesis in neurodegenerative diseases: Pathogenesis and treatment. CNS Neurosci. Ther..

[3] Cannon J.R., Greenamyre J.T. (2011). The role of environmental exposures in neurodegeneration and neurodegenerative diseases. Toxicol. Sci..

[4] Bássoli R.M.F., Audi D., Ramalho B.J., Audi M., Quesada K.R., Barbalho S.M. (2023). The Effects of Curcumin on Neurodegenerative Diseases: A Systematic Review. J. Herb. Med..

[5] Spanoudaki M., Papadopoulou S.K., Antasouras G., Papadopoulos K.A., Psara E., Vorvolakos T., Solovos E., Chrysafi M., Psallas M., Mentzelou M. (2024). Curcumin as a Multifunctional Spice Ingredient against Mental Disorders in Humans: Current Clinical Studies and Bioavailability Concerns. Life.

[6] Gitler A.D., Dhillon P., Shorter J. (2017). Neurodegenerative disease: Models, mechanisms, and a new hope. Dis. Model. Mech..

[7] Ceramella J., De Maio A.C., Basile G., Facente A., Scali E., Andreu I., Sinicropi M.S., Iacopetta D., Catalano A. (2024). Phytochemicals Involved in Mitigating Silent Toxicity Induced by Heavy Metals. Foods.

[8] Korczowska-Łącka I., Słowikowski B., Piekut T., Hurła M., Banaszek N., Szymanowicz O., Jagodziński P.P., Kozubski W., Permoda-Pachuta A., Dorszewska J. (2023). Disorders of endogenous and exogenous antioxidants in neurological diseases. Antioxidants.

[9] Iacopetta D., Ceramella J., Scumaci D., Catalano A., Sinicropi M.S., Tundis R., Alcaro S., Borges F. (2023). An Update on Recent Studies Focusing on the Antioxidant Properties of *Salvia* Species. Antioxidants.

[10] Basile G., De Maio A.C., Catalano A., Ceramella J., Iacopetta D., Bonofiglio D., Saturnino C., Sinicropi M.S. (2023). Ancient Wheat as Promising Nutraceuticals for the Prevention of Chronic and Degenerative Diseases. Curr. Med. Chem..

[11] Zia A., Farkhondeh T., Pourbagher-Shahri A.M., Samarghandian S. (2021). The role of curcumin in aging and senescence: Molecular mechanisms. Biomed. Pharmacother..

[12] Emami M.H., Sereshki N., Malakoutikhah Z., Dehkordi S.A.E., Fahim A., Mohammadzadeh S., Maghool F. (2022). Nrf2 signaling pathway in trace metal carcinogenesis: A cross-talk between oxidative stress and angiogenesis. Comp. Biochem. Physiol. C Toxicol. Pharm..

[13] Zielińska A., Alves H., Marques V., Durazzo A., Lucarini M., Alves T., Morsink M., Willemen N., Eder P., Chaud M. (2020). Properties, extraction methods, and delivery systems for curcumin as a natural source of beneficial health effects. Medicina.

[14] Mohseni M., Sahebkar A., Askari G., Johnston T.P., Alikiaii B., Bagherniya M. (2021). The clinical use of curcumin on neurological disorders: An updated systematic review of clinical trials. Phytother. Res..

[15] Kumar A., Harsha C., Parama D., Girisa S., Daimary U.D., Mao X., Kunnumakkara A.B. (2021). Current clinical developments in curcumin-based therapeutics for cancer and chronic diseases. Phytother. Res..

[16] Ji X., Tang Z., Liu H., Kang Y., Chen L., Dong J., Chen W., Kong N., Tao W., Xie T. (2023). Nanoheterojunction-Mediated Thermoelectric Strategy for Cancer Surgical Adjuvant Treatment and β-Elemene Combination Therapy. Adv. Mater..

[17] Svolacchia F., Brongo S., Catalano A., Ceccarini A., Svolacchia L., Santarsiere A., Scieuzo C., Salvia R., Finelli F., Milella L. (2023). Natural Products for the Prevention, Treatment and Progression of Breast Cancer. Cancers.

[18] Catalano A., Iacopetta D., Ceramella J., Mariconda A., Rosano C., Scumaci D., Saturnino C., Longo P., Sinicropi M.S. (2022). New Achievements for the Treatment of Triple-Negative Breast Cancer. Appl. Sci..

[19] Kali A., Charles M.P. (2024). Curcumin as a Promising Therapy for COVID-19: A Review. Glob. J. Med. Pharm. Biomed. Update.

[20] Catalano A., Iacopetta D., Ceramella J., de Maio A.C., Basile G., Giuzio F., Bonomo M.G., Aquaro S., Walsh T.J., Sinicropi M.S. (2022). Are nutraceuticals effective in COVID-19 and post-COVID prevention and treatment?. Foods.

[21] Priyadarsini K.I. (2014). The chemistry of curcumin: From extraction to therapeutic agent. Molecules.

[22] Priyadarsini K.I. (2013). Chemical and structural features influencing the biological activity of curcumin. Curr. Pharm. Des..

[23] Islam M.R., Rauf A., Akash S., Trisha S.I., Nasim A.H., Akter M., Dhar P.S., Ogaly H.A., Hemeg H.A., Wilairatana P. (2024). Targeted therapies of curcumin focus on its therapeutic benefits in cancers and human health: Molecular signaling pathway-based approaches and future perspectives. Biomed. Pharmacother..

[24] Heger M., Van Golen R.F., Broekgaarden M., Michel M.C. (2014). The molecular basis for the pharmacokinetics and pharmacodynamics of curcumin and its metabolites in relation to cancer. Pharmacol. Rev..

[25] Jeong G.S., Oh G.S., Pae H.O., Jeong S.O., Kim Y.C., Shin M.K., Seo B.Y., Han S.Y., Lee H.S., Jeong J.G. (2006). Comparative effects of curcuminoids on endothelial heme oxygenase-1 expression: Ortho-methoxy groups are essential to enhance heme oxygenase activity and protection. Exp. Mol. Med..

[26] Khayatan D., Razavi S.M., Arab Z.N., Hosseini Y., Niknejad A., Momtaz S., Abdolghaffari A.H., Sathyapalan T., Jamialahmadi T., Kesharwani P. (2024). Superoxide dismutase: A key target for the neuroprotective effects of curcumin. Mol. Cell. Biochem..

[27] Zhu Y., Xu L., Kang Y., Cheng Q., He Y., Ji X. (2024). Platelet-derived drug delivery systems: Pioneering treatment for cancer, cardiovascular diseases, infectious diseases, and beyond. Biomaterials.

[28] Jacob S., Kather F.S., Morsy M.A., Boddu S.H., Attimarad M., Shah J., Shinu P., Nair A.B. (2024). Advances in Nanocarrier Systems for Overcoming Formulation Challenges of Curcumin: Current Insights. Nanomaterials.

[29] Bertoncini-Silva C., Vlad A., Ricciarelli R., Giacomo Fassini P., Suen V.M.M., Zingg J.M. (2024). Enhancing the Bioavailability and Bioactivity of Curcumin for Disease Prevention and Treatment. Antioxidants.

[30] Ravindranath V., Chandrasekhara N. (1981). In vitro studies on the intestinal absorption of curcumin in rats. Toxicology.

[31] Ravindranath V., Chandrasekhara N. (1982). Metabolism of curcumin-studies with [^3^H] curcumin. Toxicology.

[32] Hussain Y., Alam W., Ullah H., Dacrema M., Daglia M., Khan H., Arciola C.R. (2022). Antimicrobial Potential of Curcumin: Therapeutic Potential and Challenges to Clinical Applications. Antibiotics.

[33] Vaithiyalingam M., Sumathi D.L., Sabarathinam S. (2023). Isolation and In Silico Study of Curcumin from *Curcuma longa* and Its Anti-Diabetic Activity. Appl. Biochem. Biotechnol..

[34] Dehzad M.J., Ghalandari H., Nouri M., Askarpour M. (2023). Antioxidant and Anti-inflammatory Effects of Curcumin/turmeric Supplementation in Adults: A GRADE-assessed Systematic Review and Dose–response Meta-analysis of Randomized Controlled Trials. Cytokine.

[35] Peng Y., Ao M., Dong B., Jiang Y., Yu L., Chen Z., Hu C., Xu R. (2021). Anti-Inflammatory Effects of Curcumin in the Inflammatory Diseases: Status, Limitations and Countermeasures. Drug Des. Dev. Ther..

[36] Cox F.F., Misiou A., Vierkant A., Ale-Agha N., Grandoch M., Haendeler J., Altschmied J. (2022). Protective Effects of Curcumin in Cardiovascular Diseases-Impact on Oxidative Stress and Mitochondria. Cells.

[37] Pourbagher-Shahri A.M., Farkhondeh T., Ashrafizadeh M., Talebi M., Samargahndian S. (2021). Curcumin and cardiovascular diseases: Focus on cellular targets and cascades. Biomed. Pharmacother..

[38] Ciuca M.D., Racovita R.C. (2023). Curcumin: Overview of Extraction Methods, Health Benefits, and Encapsulation and Delivery Using Microemulsions and Nanoemulsions. Int. J. Mol. Sci..

[39] Xie X., Wang H., Williams G.R., Yang Y., Zheng Y., Wu J., Zhu L.M. (2019). Erythrocyte Membrane Cloaked Curcumin-Loaded Nanoparticles for Enhanced Chemotherapy. Pharmaceutics.

[40] Jia G., Han Y., An Y., Ding Y., He C., Wang X., Tang Q. (2018). NRP-1 targeted and cargo-loaded exosomes facilitate simultaneous imaging and therapy of glioma in vitro and in vivo. Biomaterials.

[41] Wang D., Jiang S., Zhang F., Ma S., Heng B.C., Wang Y., Zhu J., Xu M., He Y., Wei Y. (2021). Cell Membrane Vesicles with Enriched CXCR4 Display Enhances Their Targeted Delivery as Drug Carriers to Inflammatory Sites. Adv. Sci..

[42] Guan X., Xing S., Liu Y. (2024). Engineered Cell Membrane-Camouflaged Nanomaterials for Biomedical Applications. Nanomaterials.

[43] Fontana F., Molinaro G., Moroni S., Pallozzi G., Ferreira M.P., Tello R.P., Elbadri K., Torrieri G., Correia A., Kemell M. (2024). Biomimetic Platele-Cloaked Nanoparticles for the Delivery of Anti-Inflammatory Curcumin in the Treatment of Atherosclerosis. Adv. Healthc. Mater..

[44] Leyva-Gómez G., Cortés H., Magaña J.J., Leyva-García N., Quintanar-Guerrero D., Florán B. (2015). Nanoparticle technology for treatment of Parkinson’s disease: The role of surface phenomena in reaching the brain. Drug Discov. Today.

[45] Agrawal M., Ajazuddin D.K., Tripathi S., Saraf S., Saraf S.G., Antimisiaris S.G., Mourtas S., Hammarlund-Udenaese M., Alexander A. (2017). Recent advancements in liposomes targeting strategies to cross blood-brain barrier (BBB) for the treatment of Alzheimer’s disease. J. Control. Release.

[46] Sinicropi M.S., Rovito N., Carocci A., Genchi G., Dushanova J. (2012). Acetyl-L-carnitine in Parkinson’s disease. Mechanisms in Parkinson’s Disease—Models and Treatments.

[47] Pringsheim T., Jette N., Frolkis A., Steeves T.D. (2014). The prevalence of Parkinson’s disease: A systematic review and meta-analysis. Mov. Disord. Off. J. Mov. Disord. Soc..

[48] Ricci S., Casalini S., Parkula V., Selvaraj M., Saygin G.D., Greco P., Biscarini F., Mas-Torrent M. (2020). Label-free immunodetection of α-synuclein by using a microfluidics coplanar electrolyte-gated organic field-effect transistor. Biosens. Bioelectron..

[49] Siddique Y.H., Khan W., Singh B.R., Naqvi A.H. (2013). Synthesis of Alginate-Curcumin Nanocomposite and Its Protective Role in Transgenic Drosophila Model of Parkinson’s Disease. ISRN Pharmacol..

[50] Bollimpelli V.S., Kumar P., Kumari S., Kondapi A.K. (2016). Neuroprotective effect of curcumin-loaded lactoferrin nano particles against rotenone induced neurotoxicity. Neurochem. Int..

[51] Kundu P., Das M., Tripathy K., Sahoo S.K. (2016). Delivery of dual drug loaded lipid based nanoparticles across the blood–brain barrier impart enhanced neuroprotection in a rotenone induced mouse model of Parkinson’s disease. ACS Chem. Neurosci..

[52] Taebnia N., Morshedi D., Yaghmaei S., Aliakbari F., Rahimi F., Arpanaei A. (2016). Curcumin-loaded amine-functionalized mesoporous silica nanoparticles inhibit α-synuclein fibrillation and reduce its cytotoxicity-associated effects. Langmuir.

[53] Rakotoarisoa M., Angelov B., Garamus V.M., Angelova A. (2019). Curcumin-and Fish Oil-loaded Spongosome and Cubosome Nanoparticles with Neuroprotective Potential against H_2_O_2_-induced Oxidative Stress in Differentiated Human SH-SY5Y Cells. ACS Omega.

[54] Sookhaklari R., Geramizadeh B., Abkar M., Moosavi M. (2019). The neuroprotective effect of BSA-based nanocurcumin against 6-OHDA-induced cell death in SH-SY5Y cells. Avicenna J. Phytomed..

[55] Salehi B., Calina D., Docea A.O., Koirala N., Aryal S., Lombardo D., Pasqua L., Taheri Y., Salgado Castillo C.M., Martorell M. (2020). Curcumin’s Nanomedicine Formulations for Therapeutic Application in Neurological Diseases. J. Clin. Med..

[56] den Haan J., Morrema T.H., Rozemuller A.J., Bouwman F.H., Hoozemans J.J. (2018). Different Curcumin Forms Selectively Bind Fibrillar Amyloid Beta in Post Mortem Alzheimer’s Disease Brains: *Implications* for In-vivo *Diagnostics*. Acta Neuropathol. Commun..

[57] Tang M., Taghibiglou C. (2017). The Mechanisms of Action of Curcumin in Alzheimer’s Disease. J. Alzheimers Dis..

[58] Barbara R., Belletti D., Pederzoli F., Masoni M., Keller J., Ballestrazzi A., Vandelli M.A., Tosi G., Grabrucker A.M. (2017). Novel Curcumin loaded nanoparticles engineered for Blood-Brain Barrier crossing and able to disrupt Abeta aggregates. Int. J. Pharm..

[59] Fan S., Zheng Y., Liu X., Fang W., Chen X., Liao W., Jing X., Lei M., Tao E., Ma Q. (2018). Curcumin-loaded PLGA-PEG Nanoparticles Conjugated with B6 Peptide for Potential Use in Alzheimer’s Disease. Drug Deliv..

[60] Huo X., Zhang Y., Jin X., Li Y., Zhang L. (2019). A Novel Synthesis of Selenium Nanoparticles Encapsulated PLGA Nanospheres with Curcumin Molecules for the Inhibition of Amyloid β Aggregation in Alzheimer’s Disease. J. Photochem. Photobiol. B Biol..

[61] MacDonald M.E., Ambrose C.M., Duyao M.P., Myers R.H., Lin C., Srinidhi L., Barnes G., Taylor S.A., James M., Groot N. (1993). The Huntington’s Disease Collaborative Research Group: A Novel Gene Containing a Trinucleotide Repeat That is Expanded and Unstable on Huntington’s Disease Chromosomes. Cell.

[62] Garodia P., Hegde M., Kunnumakkara A.B., Aggarwal B.B. (2023). Curcumin, Inflammation, and Neurological disorders: How Are They Linked?. Integr. Med. Res..

[63] Essa M.M., Moghadas M., Ba-Omar T., Walid Qoronfleh M., Guillemin G.J., Manivasagam T., Justin-Thenmozhi A., Ray B., Bhat A., Chidambaram S.B. (2019). Protective Effects of Antioxidants in Huntington’s Disease: An Extensive Review. Neurotox. Res..

[64] Labanca F., Ullah H., Khan H., Milella L., Xiao J., Dajic-Stevanovic Z., Jeandet P. (2021). Therapeutic and Mechanistic effects of Curcumin in Huntington’s disease. Curr. Neuropharmacol..

[65] Sandhir R., Yadav A., Mehrotra A., Sunkaria A., Singh A., Sharma S. (2014). Curcumin Nanoparticles Attenuate Neurochemical and Neurobehavioral Deficits in Experimental Model of Huntington’s Disease. Neuromol. Med..

[66] Iacopetta D., Ceramella J., Catalano A., Scali E., Scumaci D., Pellegrino M., Aquaro S., Saturnino C., Sinicropi M.S. (2023). Impact of Cytochrome P450 Enzymes on the Phase I Metabolism of Drugs. Appl. Sci..

[67] Gharaibeh A., Maiti P., Culver R., Heileman S., Srinageshwar B., Story D., Spelde K., Paladugu L., Munro N., Muhn N. (2020). Solid Lipid Curcumin Particles Protect Medium Spiny Neuronal Morphology, and Reduce Learning and Memory Deficits in the YAC128 Mouse Model of Huntington’s Disease. Int. J. Mol. Sci..

[68] Soler B., Fadic R., von Bernhardi R. (2011). Stem Cells Therapy in Amyotrophic Lateral Sclerosis Treatment. A Critical View. Rev. Neurol..

[69] Meamar R., Nasr-Esfahani M.H., Mousavi S.A., Basiri K. (2013). Stem Cell Therapy in Amyotrophic Lateral Sclerosis. J. Clin. Neurosci..

[70] Thomsen G.M., Gowing G., Svendsen S., Svendsen C.N. (2014). The Past, Present and Future of Stem Cell Clinical Trials for ALS. Exp. Neurol..

[71] Markov A., Thangavelu L., Aravindhan S., Zekiy A.O., Jarahian M., Chartrand M.S., Pathak Y., Marofi F., Shamlou S., Hassanzadeh A. (2021). Mesenchymal Stem/Stromal Cells as a Valuable Source for the Treatment of Immune-Mediated Disorders. Stem Cell Res. Ther..

[72] Tripodo G., Chlapanidas T., Perteghella S., Vigani B., Mandracchia D., Trapani A., Galuzzi M., Tosca M.C., Antonioli B., Gaetani P. (2015). Mesenchymal stromal cells loading curcumin-INVITE-micelles: A drug delivery system for neurodegenerative diseases. Colloids Surf. B. Biointerfaces.

[73] Yavarpour-Bali H., Ghasemi-Kasman M., Pirzadeh M. (2019). Curcumin-loaded nanoparticles: A novel therapeutic strategy in treatment of central nervous system disorders. Int. J. Nanomed..

[74] Najafi S., Najafi P., Kaffash Farkhad N., Hosseini Torshizi G., Assaran Darban R., Boroumand A.R., Sahab-Negah S., Khodadoust M.A., Tavakol-Afshari J. (2023). Mesenchymal stem cell therapy in amyotrophic lateral sclerosis (ALS) patients: A comprehensive review of disease information and future perspectives. Iran. J. Basic Med. Sci..

[75] Dolati S., Babaloo Z., Jadidi-Niaragh F., Ayromlou H., Sadreddini S., Yousefi M. (2017). Multiple sclerosis: Therapeutic applications of advancing drug delivery systems. Biomed. Pharmacother..

[76] Qureshi M., Al-Suhaimi E.A., Wahid F., Shehzad O., Shehzad A. (2018). Therapeutic potential of curcumin for multiple sclerosis. Neurol. Sci..

[77] Natarajan C., Bright J.J. (2002). Curcumin inhibits experimental allergic encephalomyelitis by blocking IL-12 signaling through Janus kinase-STAT pathway in T lymphocytes. J. Immunol..

[78] Mohajeri M., Sadeghizadeh M., Najafi F., Javan M. (2015). Polymerized nano-curcumin attenuates neurological symptoms in EAE model of multiple sclerosis through down regulation of inflammatory and oxidative processes and enhancing neuroprotection and myelin repair. Neuropharmacology.

[79] Motavaf M., Sadeghizadeh M., Babashah S., Zare L., Javan M. (2020). Dendrosomal nanocurcumin promotes remyelination through induction of oligodendrogenesis in experimental demyelination animal model. J. Tissue Eng. Regen. Med..

[80] Prusiner S.B. (1982). Novel proteinaceous infectious particles cause scrapie. Science.

[81] Prusiner S.B. (1998). Prions. Proc. Natl. Acad. Sci. USA.

[82] Abbott A. (2010). Healthy prions protect nerves. Nat. Int. Wkly. J. Sci..

[83] Lathe R., Darlix J.L. (2017). Prion Protein PRNP: A New Player in Innate Immunity? The Aβ Connection. J. Alzheimers Dis. Rep..

[84] Caughey B., Raymond L.D., Raymond G.J., Maxson L., Silveira J., Baron G.S. (2003). Inhibition of protease-resistant prion protein accumulation in vitro by curcumin. J. Virol..

[85] Lin C.-F., Yu K.-H., Jheng C.-P., Chung R., Lee C.-I. (2013). Curcumin reduces amyloid fibrillation of prion protein and decreases reactive oxidative stress. Pathogens.

